# An Efficient and Reliable Algorithm for Wireless Sensor Network

**DOI:** 10.3390/s21248355

**Published:** 2021-12-14

**Authors:** Faheem Khan, Shabir Ahmad, Hüseyin Gürüler, Gurcan Cetin, Taegkeun Whangbo, Cheong-Ghil Kim

**Affiliations:** 1 Department of Computer Engineering, Gachon University, Seongnam 13120, Korea; faheem@gachon.ac.kr (F.K.); shabir@gachon.ac.kr (S.A.); tkwhangbo@gachon.ac.kr (T.W.); 2 Department of Information System Engineering, Mugla Sitki Kocman University, Mugla 48000, Turkey; hguruler@mu.edu.tr (H.G.); gcetin@mu.edu.tr (G.C.); 3 Department of Computer Science, Namseoul University, Cheonan 31020, Korea

**Keywords:** sensor networks, multicasting, unicasting, flooding, ns-2

## Abstract

In wireless sensor networks (WSN), flooding increases the reliability in terms of successful transmission of a packet with higher overhead. The flooding consumes the resources of the network quickly, especially in sensor networks, mobile ad-hoc networks, and vehicular ad-hoc networks in terms of the lifetime of the node, lifetime of the network, and battery lifetime, etc. This paper aims to develop an efficient and reliable protocol by using multicasting and unicasting to overcome the issue of higher overhead due to flooding. Unicasting is used when the desired destination is at a minimum distance to avoid an extra overhead and increases the efficiency of the network in terms of overhead and energy because unicasting is favorable where the distance is minimum. Similarly, multicasting is used when the desired destination is at maximum distance and increases the network’s reliability in terms of throughput. The results are implemented in the Department of Computer Science, Bacha Khan University Charsadda (BKUC), Pakistan, as well as in the Network Simulator-2 (NS-2). The results are compared with benchmark schemes such as PUMA and ERASCA, and based on the results, the performance of the proposed approach is improved in terms of overhead, throughput, and packet delivery fraction by avoiding flooding.

## 1. Introduction

Nowadays, designing an efficient WSN is an important research area. A WSN is a network of components that transfer the collected information from source to destination wirelessly. Collected information is forwarded with a gateway through multiple nodes to the other group/network such as wireless ethernet. In the presence of a wireless network, WSNs can be implemented/organized in places where they are costly to be deployed and can be used in many applications such as intelligent transport system, smart agriculture, military application, disaster recovery, wildlife monitoring, community networking, vehicular computing, etc. [1,2,3]. A WSN consists of sensor nodes with limited memory, CPU power, and energy capabilities. Sensors run different applications for different purposes such as tracking, localization, monitoring and event detection. These applications are updated and configured over the mesh or group of nodes continuously.

In WSN [4], data are communicated through multicasting, unicasting, broadcasting, any casting, and flooding, but the discussion is limited to multicasting, unicasting and flooding in this paper. In multicasting and unicasting, reliability decreases due to regular changes in the topology because only a particular route exists between the receiver and a source but at the cost of less energy consumption [5,6,7]. On the other hand, flooding distributes the data in the group immediately, as the data are received by any mesh member, which gives reliability at the cost of higher overhead to the group and the network [8,9]. Overheads are the resources utilized by every sensor node such as computation time, bandwidth, energy and memory, etc. Likewise, throughput is the data transmission per second through the network. A flooded network becomes congested soon by increasing the overhead, and as a result, a packet will be in a queue. Due to a large queue, the throughput decreases [10,11,12,13]. Unicasting, multicasting and flooding protocols in the wireless sensor network are always studied in respect of sender-based and receiver-based routing. Examples of receiver-initiated factors are protocol for unified multicasting through announcement (PUMA) [14], core assisted mesh based protocol (CAMP) [15], and efficient and reliable core assisted multicast routing protocol in MANET (ERASCA) [16]. Sender based protocols are dynamic core based multicasting protocol (DCMP) [17], neighbor supporting multicast protocol (NSMP) [18] and on-demand multicast routing protocol (ODMRP) [19].

The recent research in multicasting and unicasting based routing protocols in the wireless network and wireless sensor network motivates the researchers in hybrid protocols. In hybrid protocols, unicasting and multicasting can be used in the same protocols according to their need. This hybrid approach provides efficiency in overhead and energy utilization more effectively with a longer lifetime of the node and the network. Hence, node/group/network efficiency is essential for achieving efficient communication as compared to flooding, where the node and the network lifetime decrease due to frequent flooding. In addition to efficiency, a stable protocol is necessary during high mobility in the WSN’s environment. The high mobility and harsh channel conditions deteriorate the protocol’s reliability in terms of throughput in unicasting and multicasting. It is, therefore, necessary to evaluate the reliability of the protocol during unicasting and multicasting. An efficient and reliable core assisted multicast routing protocol in MANET (ERASCA), CAMP and PUMA were used previously in MANET [11]. In these protocols, flooding is used inside the mesh/receiver group to transmit data packets to the destined receiver. This increases the reliability of the packet transmission with the increase in the overhead. However, ERASCA protocol efficiency and reliability can be further improved using a hybrid approach in the proposed extended ERASCA protocol (EERASCA).

In the proposed EERASCA, a hierarchy is created in the network using multicasting and unicasting. Unicasting is used when the desired destination is at minimum distance, and multicasting is used when the desired distance is at a significant distance irrespective of the flooding where distance did not consider. This approach can then coordinate the transmission to reduce the overhead enormously by avoiding continuous flooding. The proposed protocol increases the efficiency of the cluster/ group/network in term of overhead and energy utilization and reliability in term of throughput. This efficiency and reliability improve the lifetime of the cluster head/core duration, group member duration and network duration.

The organization of this paper is as follows: In Section 2, related work is explained in detail using mesh and tree-based protocols. In Section 3, the proposed methodology is explained by using multicasting and unicasting instead of flooding within the mesh of a group. In Section 4 and Section 5, the formation of the receiver group and formation of the mesh is explained diagrammatically with examples. In Section 6, the proposed algorithm for data forwarding is explained in detail using multicasting and unicasting. In Section 7, performance evaluation is examined in detail by using NS-2 as well as implemented in real-time in the computer science lab of BKUC. Finally, Section 8 concludes the paper and shows the improvement in performance by using multicasting and unicasting.

## 2. Related Work

According to a literature review in receiver-initiated mesh-based routing [13], flooding is used within the mesh of a group to transmit the packet to the desired destination. This increases the robustness of the network by utilizing more overhead. To overcome the issue of flooding, multicasting and unicasting are the favorable approaches in WSN.

The IP multicast service model is used in ERASCA protocol [16] to allow a sender to transmit its data to the receiver group without any previous record/communication with the particular group. In this protocol, the sender is not essential to become part of the group for data transmission. Likewise, the core node is responsible for forming, maintaining, and updating the receiver group through status declaration (SD) message. The connectivity list in ERASCA is formed through SD message, in which all members of the group are connected through intermediate nodes. All the member of the receiver group and forwarding nodes jointly form the mesh through SD message. When the data are received by the mesh member through sender then it is immediately flooded inside the mesh to reach the desired destination.

PUMA [14] is using an IP multicast service model for data forwarding within the mesh. It is a receiver-initiated mesh-based protocol and uses the concept of core node to form, maintain, and update the group. The group nodes and intermediate nodes collectively form the mesh. In PUMA, when the source node transmits the packet from the sender to the destined receiver, it passes within the network and reaches the mesh. When the mesh member picks the data, it is flooded inside the mesh to reach the desired destination.

ODMRP [19] is a sender-initiated mesh-based routing protocol. The concept of forwarding group for data transmission is used in ODMRP. The idea of the core node is used in ODMRP for the update and maintenance of the group. In ODMRP, the data are transferred from the sender to the destined receiver; then, the sender receives an acknowledgement for the successful reception of the data. On the other hand, if a sender does not entertain ACK, the packet will be transmitted again. This retransmission decreases the efficiency and reliability in terms of overhead, usage of energy and throughput. Likewise, CAMP, AODV [20] and efficient zero-control-packet broadcasting for mobile ad hoc networks (ECHO) [21] are sender-initiated routing protocol and use flooding within the mesh for successful communication.

AWFCC [22] is a congestion control algorithm based on clustering approach to improve energy consumption in a wireless sensor network. In AWFCC, nodes are clustered by greedy first search algorithm and packet forwarding are performed through ant colony optimization based routing. This algorithm performs well in low mobility situation but during high mobility cluster head failure situation increases and it starts fresh reconfiguration continuously for the next cluster head. This frequent reconfiguration for the next cluster head effecting its efficiency in term of energy, throughput and extra overhead. However, in ERASCA and EERASCA protocol mirror core is already selected and as soon as the core node (cluster head) failure occur then the mirror core become the core node and the continue the responsibility as a core node without any delay and reconfiguration. 

FUCARH [23] is used to increase the lifetime of the wireless sensor network by using inter-cluster routing and cluster maintenance approach. In this method, a cluster head is selected through fuzzy logic efficiently and then divide the network into unequal clusters. The unequal clusters are divided on the bases of the remaining energy, distance of cluster head from the neighbors and base station. This protocol fails in situation when the mobility increases because due to increase in mobility the size of the cluster also changes frequently and hence increases the complexity. In addition, due to the inter-cluster approach when the end receiver of each cluster came in the range of two cluster head then the movement of nodes within the clusters increases due to shifting of nodes from large cluster to small cluster and hence increases the node exchange.

This paper [24] also uses the mesh approach for data forwarding. In this approach, as soon as the data packet is received from the sender to the group member then it immediately floods the packet within the group to reach to the desire destination. As a result, the flooding increases the overhead and decreases the lifetime of the network. However, in the proposed approach, the flooding is avoided to decrease the overhead and increases the life time of the network.

All the protocols use the internet protocol (IP) service model for allowing the sender in the network to send its data to the multicast group [16]. In the Modified ERASCA protocol, a bootstrapping is used where a node makes a multicast group and starts a periodic broadcast message in a network, called SD or hello message. Through the SD message, a group is periodically flooded at a regular interval by the core node and forms the network’s connectivity list. The connectivity list forms the best possible route in a network as well as in the mesh. After reaching the data inside the mesh, it will ultimately reach the desired receiver in the group.

In the aforementioned approaches, flooding is used immediately when any mesh member of the group receives the data. After flooding, the data reaches the desired receiver. This flooding creates overhead and congestion, which stage the network’s resources and lifetime [25,26]. In the proposed EERASCA, multicasting is used for a distant destination, and unicasting is used for the nearest node within the mesh for data transferring to the desired receiver, which increases the efficiency and reliability in terms of overhead, network lifetime and battery consumption as compared to benchmark protocols such as PUMA and ERASCA.

## 3. Proposed Methodology

### 3.1. Overview

In this paper, an improved and extended version of ERASCA is proposed, known as the Extended ERASCA protocol (EERASCA). However, there are few similarities between the previous protocols and the proposed protocol, as shown in Table 1. The stages which are similar in both approaches are discussed shortly. However, Stages 3 and 4 are repeated and explained in more detail to understand the paper correctly. However, Stage 7, Stage 8 and Stage 9 are modified in the proposed protocol and discussed in more detail.

### 3.2. Proposed Protocol

**Figure 1:**
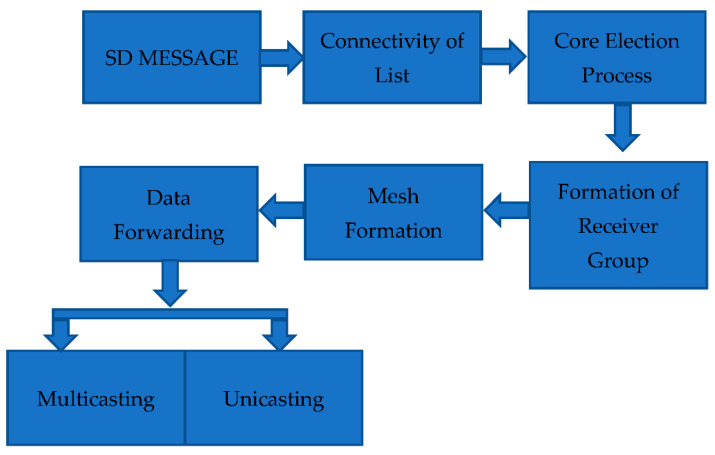
Block Diagram of the Proposed Protocol.

The proposed model of EERASCA is shown in Figure 1 and it is implemented by using the SD message, connectivity of lists, the core election process, receiver group formation, mesh formation, data forwarding process through unicasting and multicasting, an algorithm of the proposed EERASCA and finally the implementation of the protocol.

EERASCA is a receiver-initiated mesh-based algorithm. At the start of the communication, the first receiver that wishes to include in the receiver group will broadcast SD message to the neighbors. If any group exists, then the receiver will receive the group confirmation reply. However, if no group existed, then the receiver will announce himself as a group leader and start broadcasting a message about the group’s formation. This receiver node is known as a group leader or core node, and now it forms a connectivity list. Through connectivity lists, nodes are connected and transmit and receive the data from the source node. Through SD message, all the nodes are aware of other nodes location and battery capacity within the receiver group.

At the start of the communication process, the first node that starts the communication is selected as a core node by default. However, after the core failure, the group members shared the SD messages with all the interested receiver and the receivers that want to be the member of the group will form the receiver group. The group will contain the receivers with similar interest. Now all the receivers will conduct an election for the core node, and the most suitable receiver having the maximum battery capacity and suitable location (within the center of the group) is selected as a core node. Now it is the responsibility of the core node to maintain and update the receiver group. After the receiver group formation, the mesh formation will continue, and it is explained in detail in Section 5.

In the previous protocol, the data are forwarded within the mesh through flooding, but the data are forwarded within the mesh through unicasting and multicasting in the proposed protocol. The proposed protocol improves the reliability and efficiency in term of throughput and overhead by avoiding flooding. The proposed algorithm is implemented through NS-2. The experimental implementation of EERASCA assures the superiority over ERASCA in term of overhead, PDF and throughput.

### 3.3. Core Election Process

Core election process affect the performance of the routing scheme because inappropriate core election procedure declines the network performance in term of network resources. Traditionally, random based selection and connectivity-based selection approaches select the core of the group. In the former approach, the core is selected on random generated number to begin the communication for specific group. The core is selected without any predefined parameters and pre-defined information. Hence, the node with a less battery energy, a smaller number of connected nodes and with inadequate position can be nominated as core node. This inadequate selection of the core node will increase the core failure and hence increases the reconfiguration process. This increase in the reconfiguration process increases the overhead, congestion, packet drops and network resources quickly. To resolve the issue in random based approach, connectivity-based approach is introduced, in which the core node is elected only on number of connected neighbors. hence a node with appropriate position is selected as core node for the group. The problems occur in this approach when the group sizes increase. This increases in the group size will exhaust the energy of the core node quickly and again the failure of the core node increases and hence increases the reconfiguration process. In both the approaches, the frequent core failure affect the QoS based applications. However, in this approach the core is selected on multiple parameters such as battery capacity, sequence number, number of connections and position. Hence, the core with best position and maximum battery capacity is selected as a core node with the increase in the lifetime of the core node. this will also reduce the core failure and hence decreases the re-election process for another core. 

In core election process, nodes “*n*” is equally distributed using random topology with the velocity of 0-Vmax. Initially two conditions occur. First, if *n* wishes to become a member of the already existed group, then it will join the group having their own core node because the existed core node was continuously broadcasting the SD messages within the receiver group. The SD messages are further broadcasted by the group member to reach to every node within the vicinity of the group. These messages are received to node *n* through any group member m. Hence, node *n* joins the group as defined in the SD message and actively participating tin the group. Secondly, in the absence of the receiver group, the node *n* will declare himself as a core node and broadcasting the SD message as a core node to inform others node to join the group. After formation of the receiver group, the formation of the mesh is started as shown in Section 5. 

**Figure 2:**
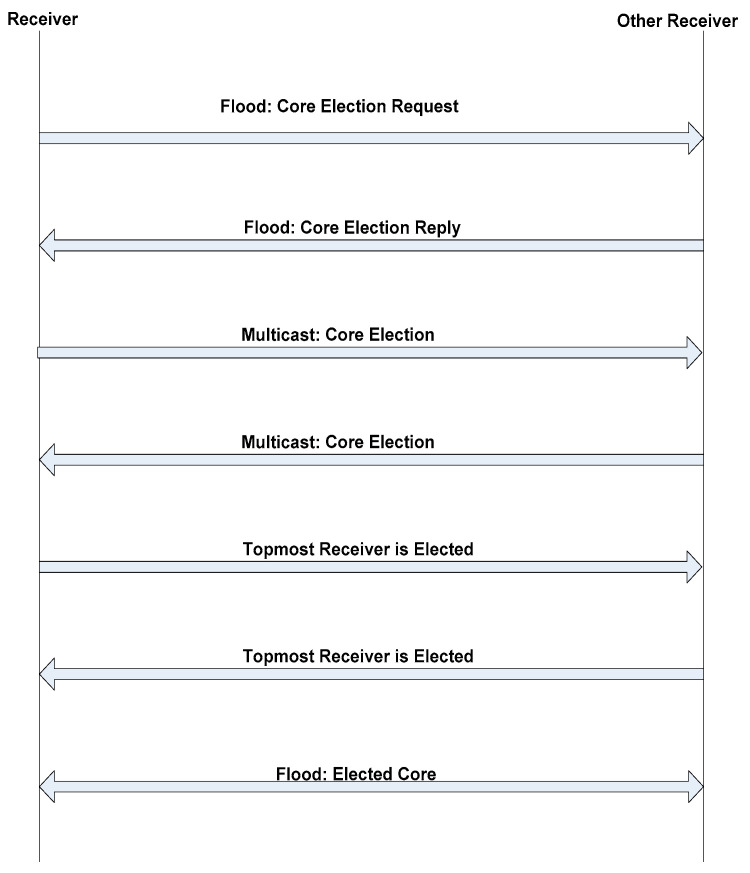
Core Election Process.

Now when the core node fails then the proposed core election will start. the members of the group will be aware from the core failure announcement (CFA). The CFA is communicated within the group members through GR and IR if GR and IR did not receive the SD within the predefined threshold i.e., three consecutive SD messages. After the core failure, a core election message (CEM) is broadcasted in the group to select the best possible receiver as a core node in term of battery capacity, sequence number, number of connections and position. Now to find out the best possible core node a core election request message is broadcasted in the group. In reply, the group members will response through core election reply message. Now all the group members will be aware from the status of each other in term of battery capacity, sequence number, number of connections and position as a result a best possible node is selected as a core node. The core election process is shown in Figure 2.

## 4. Formation of Receiver Group 

The basic principle of EERASCA is the way of communication through which data are transferred between each node in the group. It should be known that the network is a collection of all the nodes within the predefined range, but the group is the collection of nodes that have a common interest. For communication in a group, the most appropriate method is multicasting, which increases the efficiency of the network by reducing overhead. On the other hand, communication in ERASCA within the group is performed through flooding, which increases the reliability but with a larger overhead. 

Two cases are used during the receiver group formation. Firstly, when a node n is willing to be a part of the current receiver group. If node n receives any request from the group member, it will transmit a join request for the membership of the receiver group. In response, a join ACK is communicated to n by the group member. It joins the group and begins sending messages that identify the group. Secondly, if no receiver group exists, then the first receiver that joins the group will declare itself as a core node and begin disseminating SD messages as a core node to notify the receivers to join the group. After joining the group, the group’s receivers continuously checked the periodic updates of the SD messages, and if not received within 2× SD interval, then it is considered that the core node failure has occurred. A receiver announces a core failure request (CFR) for the confirmation of the core node failure.

In contrast, if the core failure does not occur, the CFR communicates a receiver that accepts a new SD message than the sequence number in the CFR. The receiver then communicates the fresher sequence number to the receiver group, which relies on a similar path to the sender that started the CFR request. If the core failure occurs in reality, then the CFR request will not reach the sender because of the disconnection of the connectivity list. Therefore, the core failure announcement (CFA) should be fixed to a definite time interval which is enough for the CFA Request to send back to the receiver which started the CFA request.

## 5. Formation of Mesh

**Figure 3:**
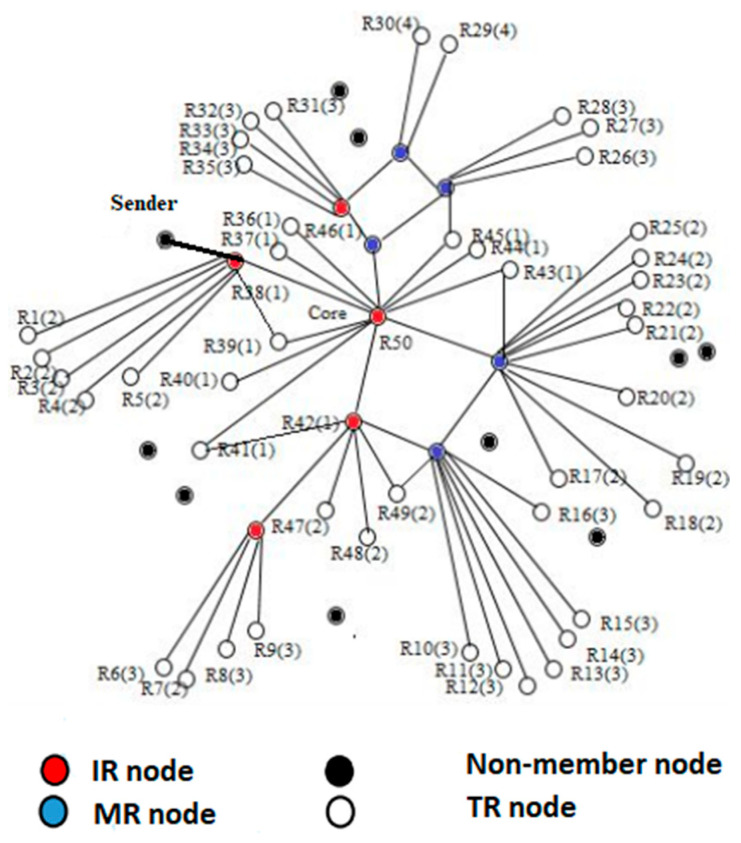
Mesh Formation.

The nodes in the network are characterized as non-group (NM) members and group members. The former is not part of the mesh and represented in a black dot, as shown in Figure 3. The latter is divided into mesh relay (MR), intermediate receiver (IR) and terminal receiver (TR). The MR is the intermediate node between the core and a receiver and represented as a blue color. The TR is known as a terminal receiver, and the mesh finish on it, TR does not contribute to the packet forwarding and is represented in white color. Likewise, the IR acts as an intermediate node and a receiver and are represented as a red dot. The intermediate node between R48 and the core node is R42 node. At the start of the communication, only receivers are mesh members, but later on, the MR will also be a part of the mesh member because they occur between the TR and core node and communicate the data. In the PUMA and CAMP, the data are transmitted through flooding within the group. When the data are transmitted from a source to any mesh member, it is flooded immediately inside the mesh. As a result, the desired receiver within the group receives the data through flooding. The packet received twice by any group member will be rejected because the information about the repeated packet is already saved in the cache, which will further waste the resources of the group member. In Figure 3, R50 is the parent node of R38. The affiliation of the parent to child remains until it reaches the group member R38. When it reaches R38, then it immediately floods within the mesh of a group. Moreover, if the number of group members increases, then flooding is further worsening the performance. 

## 6. Data Forwarding in Extended ERASCA Protocol (EERASCA)

**Figure 4:**
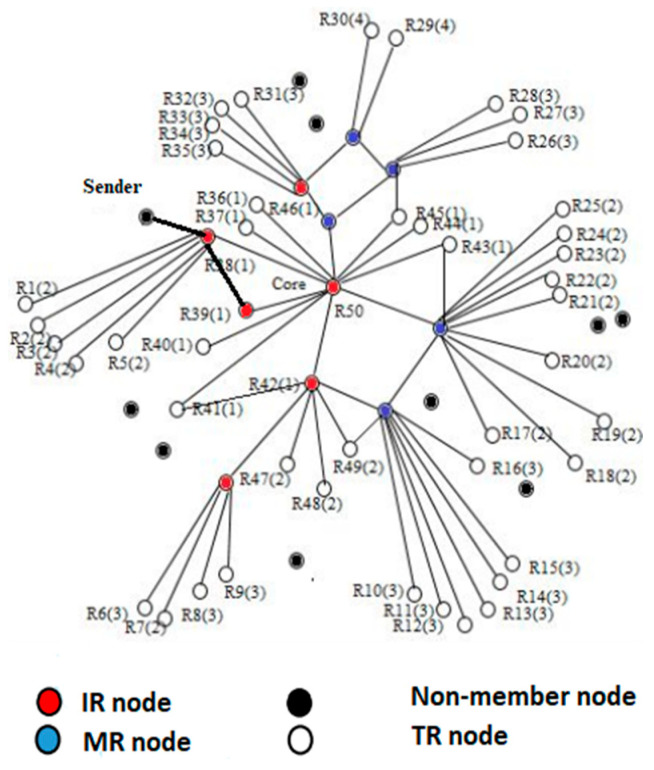
Data Forwarding in EERASCA.

In this section—Stages 7, 8 and 9 are explained along with mathematical analysis. In EERASCA, it is the responsibility of the member node to transfer the data packet to the desired node either through multicasting or unicasting (Stage 9). For example, as shown in Figure 4, a sender wants to send data packet D to R39. However, the source is not part of the mesh and will transfer the data packet to R39 through mesh member. In this situation, R38 is in a significant position, and it is the responsibility of R38 to transfer the data from the sender/source within the mesh to the desired destination receiver. For this purpose, a routing table is maintained through SD message. The core broadcasts the SD message periodically to maintain and update the group, and all the mesh members will be able to know their connected members through a SD message. Through the routing table, R38 will be able to transfer data packet through unicasting or multicasting. 

Two situations arise in EERASCA (Stage 7): (1) If the destined receiver from R38 is within the three-hops distance as shown in Figure 4, then unicasting is used for data forwarding to quickly receive the data with less overhead because unicasting creates less overhead as compared to flooding. (2) If the destined receiver is at a four-hop distance or more, then multicasting is used compared to flooding. Again, multicasting is creating less overhead and sends the data only to desired nodes. Hence, unicasting and multicasting create less overheads and less delays as compared to flooding.

Here, n represents the number of hopes, and it is equal to three. Hence, if the destined node receives within the n hopes distance (represent less distant nodes), unicasting will be used. However, if n+1 (represent far away distant node) occurs, then multicasting will be used. In ERASCA, the packet is reached to R38 from a source outside the mesh, then it is flooded in the mesh, and the destined receiver receives the data. As a result, all the nodes receive the packet and resources of the group and network decreases.

This section is not mandatory but may be added if there are patents resulting from the work reported in this manuscript.

### 6.1. Mathematical Analysis for Maintenance of Overhead 

Let assume that “*µ*” represents link failure rate due to mobility. For the maintenance of path between the mesh members the effect of overhead is calculated which are as follows:

- Receiver is not within the radio range of its current (x) IR or MR; (y) transfer within its neighboring IR or MR.
- The secondary core, i.e., mirror core is out of the radio range of its (x) current IR or MR

Core node is not accessible to mesh members. 

The probability for EERASCA is calculated for the scenarios discussed above. The messaging overhead is evaluated for the maintenance of mesh. 

**Figure 5:**
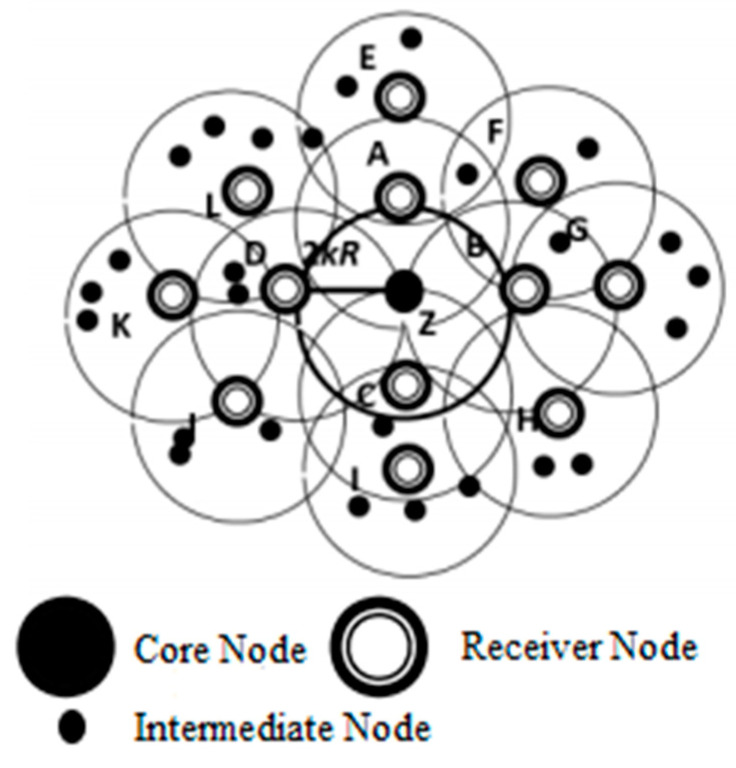
Density of Clusters with 2kR Distance [4].

In Situation 1 (x), the overhead of messaging is 2k×2 as shown in Figure 5, where k represents hop and r represents the radio range. In this scenario, the receiver informs the current IRMR about the status. In addition, after joining a new group it informs the current IRMR and its neighboring IRMR. The probability of exiting IRMR is $\frac{\left(2k-1\right)}{k^{2}}\times ¥,$ depends on the following derivation:

On the edge of the group, periphery receivers are $\;dk^{2}-d{\left(k-1\right)}^{2}=d\;\left(2k-1\right)$ and average of IR and MR is dk2 up to k-hop. The probability of exiting receiver from IRMR is $\frac{d\left(2k-1\right)}{dk^{2}}$ and that of the periphery receiver to exit from IRMR up to total receivers are $\frac{d\left(2k-1\right)\;IRMR}{R}\;$=$\;\frac{d\left(2k-1\right)}{dk^{2}}$.

¥ represents the probability of uncovered area of the mesh where receiver exit, which is shown as: $\frac{size\;of\;all\;IRMR}{size\;of\;covered\;area}\;$=$\;\frac{\pi {\left(kr\right)}^{2}\times IRMR}{area}$

(1) $$¥=\{\begin{array}{c} (1-\frac{\pi {\left(kr\right)}^{2}\times IRMR}{area}),\;\;\;if\;\frac{\pi {\left(kr\right)}^{2}\times IRMR}{area}<1\\ 0,\;otherwise \end{array}$$

In 1 (y), the messaging overhead is 2k and it is used to inform the current and previous neighbor. It concludes that the receiver is moving between the neighborhoods of the covered area. Hence, the moving receiver probability is $\frac{\left(2k-1\right)}{k^{2}}$ × 1 − ¥.

In 2 (x), the messaging overhead is 2k×2 and the departure probability of mirror core to its current MR and IR is $\frac{\left(2k-1\right)}{k^{2}}\times ¥$. The messaging overhead is 2k because mirror core informs the primary core and IRMR, about its mobility and its probability which is $\frac{\left(2k-1\right)}{k^{2}}$ × 1 − ¥.

In C, the primary core is heading towards the edge because of its mobility and such movement of the core node is tackled by Scenarios 1 and 2. The probability of orphanage receiver, when the core node leaves the mesh is $\frac{2}{dk^{2}}$. The messages for the new core are represented as $\frac{1\;}{2}dk^{2}-1$. To update the receiver group 2k×dIRMR messages are used. When the receiver leaves the mesh of a group then the average number of messages are:

(2) $$\mathrm{Messag}e_{\mathrm{Receiver}}=\;2[4k\times \frac{\left(2k-1\right)}{k^{2}}\times ¥\;+2k\times \frac{\left(2k-1\right)}{k^{2}}\times \;1-\;¥]\;\mathrm{Taking}\;\mathrm{common}\;2k\;\frac{\left(2k-1\right)}{k^{2}}\;\mathrm{Messag}e_{\mathrm{Receiver}}=2[2k\;\frac{\left(2k-1\right)}{k^{2}}\;(2¥+1-¥)]\;\;\mathrm{Messag}e_{\mathrm{Receiver}}=\frac{4\left(2k-1\right)}{k}\;(¥+1)$$

Equation (2) shows the overall messages created by the receivers of the group.

Similarly, the overhead created for the reconfiguration of the next core, which are as follows:

(3) $$\mathrm{Messag}e_{\mathrm{Core}}=\frac{2}{dk^{2}}\left[\left(\frac{1\;}{2}dk^{2}-1\right)+\left(2k\times d_{IRMR}\right)+4k\times ¥+2k\times \left(1-\;¥\right)\right]\;\mathrm{Messag}e_{\mathrm{Core}}=\frac{2}{dk^{2}}\left[\left(\frac{1\;}{2}dk^{2}-1\right)+\left(2k\times d_{IRMR}\right)+2k\;\left(2¥+1-\;¥\right)\right]\;\mathrm{Messag}e_{\mathrm{Core}}=\frac{2}{dk^{2}}\left[\left(\frac{1\;}{2}dk^{2}-1\right)+\left(2k\times d_{IRMR}\right)+2k\;\left(¥+1\right)\right]$$

To maintain the mesh in Equation (4), the average number of messages for combining $\mathrm{Messag}e_{\mathrm{Core}}$ and $\mathrm{Messag}e_{\mathrm{Receiver}}$ are required which are as follows:

(4) $$\mathrm{Messag}e_{\mathrm{ERASCA}}=\frac{2}{dk^{2}}\left[\left(\frac{1\;}{2}dk^{2}-1\right)+\left(2k\times d_{IRMR}\right)+2k\;\left(¥+1\right)\right]+\frac{4\left(2k-1\right)}{k}\;(¥+1)$$

### 6.2. Mathematical Analysis of Overhead (AO)

The following three steps are followed in EERASCA:

Step 1: At the beginning, the data should be moved from the source towards the mesh, the source node then forwards the data to IRMR of k hop. In the case of multicasting, the number of nodes in the group should be n+1, however, in unicasting it should be n. 

Step 2: When the transmitted data are reached to the mesh member, it is immediately multicasted/unicasted towards the IRMR. 

Step 3: After multicasting/unicasting, the desired receiver receives the data. Suppose that the mesh IRMR is a fraction *µ* and in the multicast group when there are n members. Hence, IRMR within the mesh is μ×IRMR. Likewise, several receivers (*n*) are equally distributed in the IRMR, and (1−μ) is on the edge of IRMR. Hence, the transmission rates for packet forwarding within EERASCA are:

(5) AO=k+n+1×(IRMR−1)+k×(1−μ) n+k×μnAO=k+n+1×(μIIRMR−1)+kn−kμn+k μnAO=k+n+1×(μIRMR−1)+kn

### 6.3. Algorithm of EERASCA 

Depending on the two situations, a mechanism of unicasting and multicasting is used in this paper. The Algorithm 1 (Stage 8) contains four steps which are as follows:
**Algorithm 1.** The Proposed Algorithm of EERASCA./******the initialization phase******/1 Broadcast “SD message” about connected neighbors from core node2 Receiving of “SD message” about the neighbors /****** The running phase******/3 while (receive a data packet D by Mesh member M)4    Calculate the connected neighbors of M5     Update the routing table of Mand assume that neighbors are represented by G6     if (G ≤ 3) then7 Cancel the multicasting of M;8     else9    update the routing table of M;10     if (G ≥ 4) then11 multicast M;12 Break from while loop;

(1) Initially, every node generates its SD message about the status of its position within its neighbors.
(2) When mesh member, e.g., R38, broadcasts the SD message within its neighbors, then the node at three-hops distance will be stored in its routing table.
(3) The routing table is updated, and Data D is transferred to R39 through unicasting.
(4) Let us suppose that R51, if the node is not within the three-hop distance, then the Data D will be transferred through multicasting.

## 7. Result Comparison and Performance Evaluation

In this experiment, wireless motion sensors are used. The motion sensor is divided into a base station and a sensor. 

**Figure 6:**
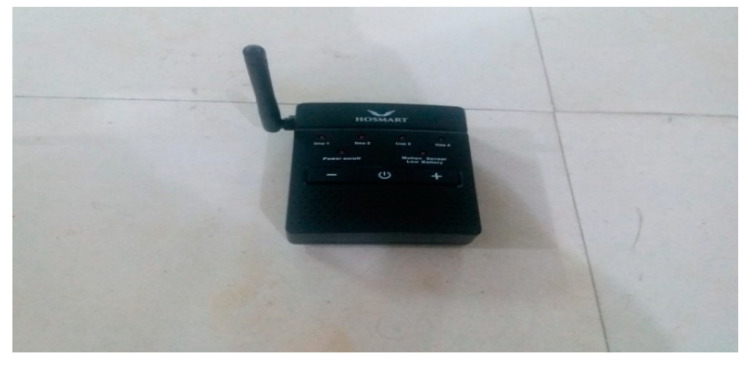
Base Station.

The base station is shown in Figure 6 consists of an antenna and AC adapter with 4AA batteries, LED indicator for power on/off, low battery LED indicator, 4 receiver LED indicator, power and volume. The antenna covers an area of 1/4 miles. Hence, nodes in this defined radio range are entertained and communication takes place within this area. The AC adaptor is used for indoor detection. However, in remote places such as mountains and battlefield scenario and vehicle detection, 4AA batteries can also be used. LED indicator shows whether the power is on or off. Likewise, when the battery is low, then LED light is ON, which indicates the recharging. Each base station can entertain four receivers at a time, and there are four LED lights on each receiver. However, the number of a receiver can be increased by connecting the base station with each other.

**Figure 7:**
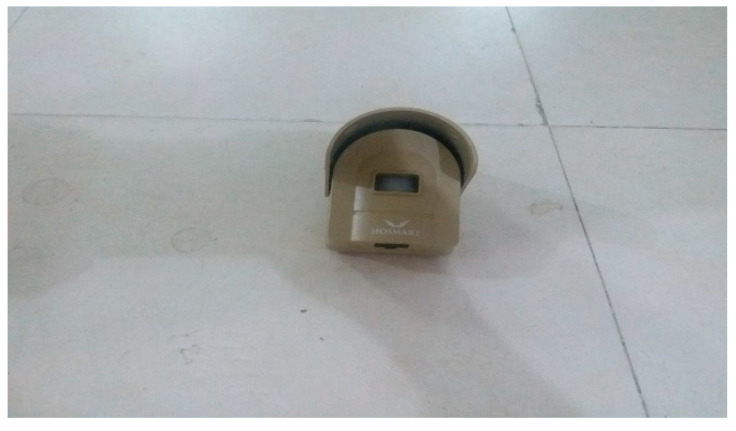
Sensor.

The sensor as shown in Figure 7 is consisting of Sensor Eye, Switch 1 and Switch 2. Sensor Eye is used for the detection of nodes. Switch 1 is composed of HI, MID1, MID2 and Low. In HI mode the detection range is 30 ft. Likewise, the detection ranges for MID1, MID2 and Low are 25 ft, 20 ft and 15 ft consecutively. The detection range for small and large areas is defined for the need of the user and situation. Switch 2 having the capability to form four cluster head, i.e., CH1, CH2, CH3, CH4. Each cluster head defines Zone 1 to Zone 4, i.e., its own zone. The sensor having 18,650 batteries, when fully charged, will operate for up to 1 year.

### 7.1. Real-Time Experiment Setup

**Figure 8:**
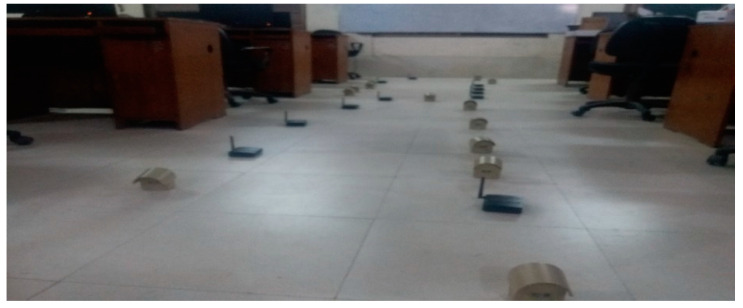
Different Scenarios performed in the Computer Science Lab, Bacha Khan University, Charsadda.

This paper estimates, executes and relates the proposed protocol with some well-known protocols such as ERASCA and PUMA. The following metrics such as mobility, number of receivers and covered area are used with subsequent factors as shown in Table 2. Three scenarios in Figure 8 are considered in terms of PDF, overhead and throughput. Based on these scenarios, conclusions are made that the proposed protocol is more efficient in term of overhead and reliable in term of PDF and throughput. 

Scenario 1: Here, the number of receivers is 40 with 30 × 30 m^2^ covered area. Here mobility changes from 0 to 4 m/s with 1 m/s intervals.

Scenario 2: Here, the receivers vary from 10–40, and the mobility is fixed at 4 m/s with a 30 × 30 m^2^ covered area.

Scenario 3: Covered area varies from 0 × 30 m^2^. Here the number of receivers is 20 and mobility 4 m/s.

### 7.2. Protocol Evaluation 

The following metrics, i.e., throughput, PDF and overhead are used in this experiment as shown in Table 2. Throughput is the amount of data packet transmission from source to destination at a specific interval and PDF is the amount of data packet received divided by the transmitted data. Finally, overhead is the amount of total packet send divided by the total packet received.

#### 7.2.1. Scenario 1

**Figure 9:**
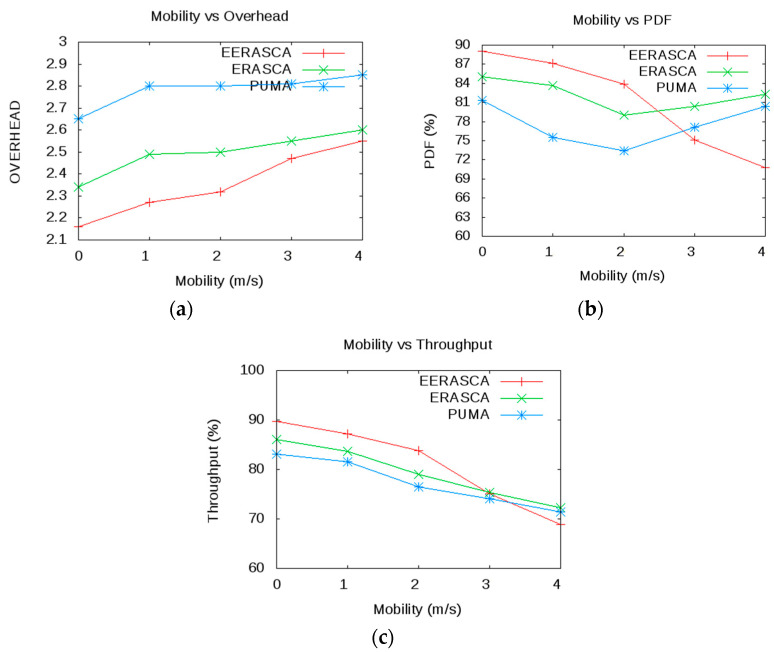
Comparison of Mobility with Overhead, PDF and Throughput.

In Scenario 1, the mobility varies from 0–4 m/s, and all other parameters are fixed, as shown in Table 2. Based on these parameters, several results are obtained from ERASCA and PUMA, and a comparison is made with the EERASCA. As shown in Figure 9a, when the mobility is low, then EERASCA transfers 90% packets, whereas the packet transfer rate of ERASCA and PUMA are 86% and 82% consecutively. As the mobility increases, then the PDF drops due to frequent topology changes. In high mobility, the packet drops increase; this decreases the PDF with the increase in overhead, as shown in Figure 9b. The high mobility increases the overhead and link failures, and hence the retransmission of packets increases. This retransmission of packet increases the flooding and hence decreases PUMA and ERASCA in terms of overhead. In Figure 9c, throughput decreases due to an increase in overhead. 

Throughput is the data transmission per second, and the increase in overhead increases the congestion in the network. This increase in congestion limited the capacity of the actual bandwidth. For example, the actual bandwidth is 3 Mbps, and the end receivers can only handle 1 Mbps due to congestion, which limited the data transmission to 1Mbps, which is the wastage of resources in terms of bandwidth. Hence, the throughput is 1/3 of the bandwidth. In Figure 9, EERASCA shows less performance in high mobility in terms of throughput and PDF than ERASCA and PUMA. In high mobility, unicasting and multicasting do not give promising results due to regular topology changes and link failure. On the other hand, ERASCA and PUMA show improvement in throughput and PDF but with higher overhead and his higher overhead is due to continuous flooding. The confidence interval for PDF, overhead and throughput is shown in Table 3 in the presence of mobility (0–4 m/s), which shows the mean value, highest and lowest value.

#### 7.2.2. Scenario 2

**Figure 10:**
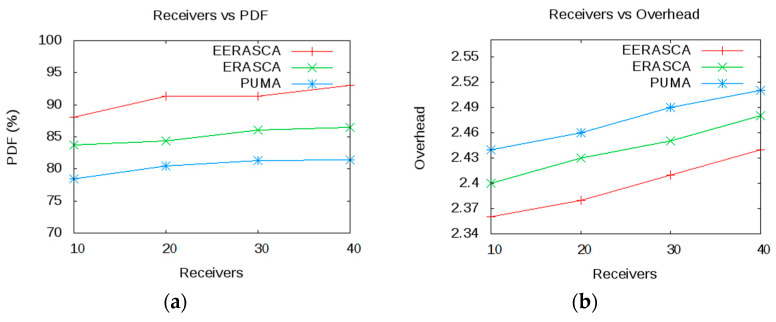

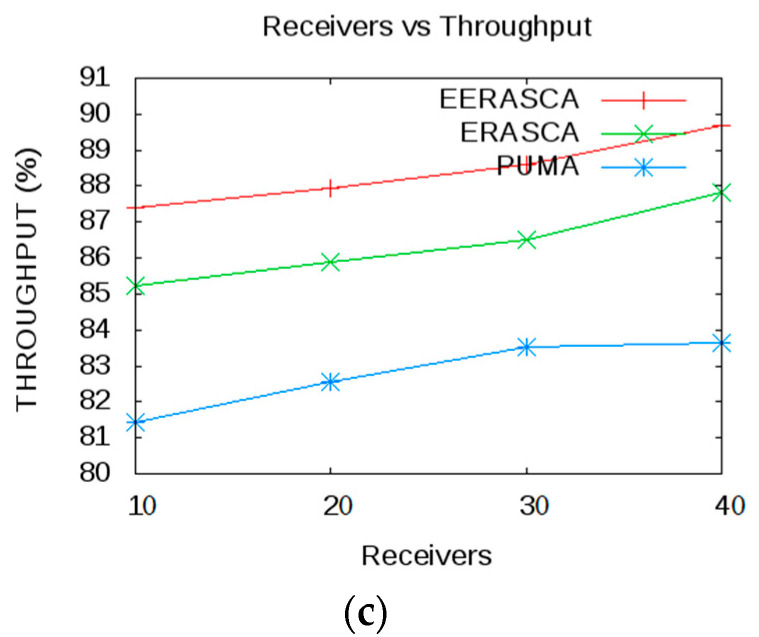
Comparison of Number of Receivers with PDF, Overhead and Throughput.

Figure 10 shows that by increasing the receivers then the performance of overhead, PDF and throughput are improving in EERASCA as compared to PUMA and ERASCA. Figure 10a,b shows that the increase in PDF and throughput is due to the presence of many alternate paths between the receivers and the group members which minimizes the packet loss as compared to longer and fewer paths. Packet loss is caused due to data transmission error and congestion. Packet loss arises when one or more data packets are moving across a network fail to reach the desired destination. Hence, as the number of receiver’s increases, then there will be richer connectivity between the nodes in the group and increases the PDF and throughput. 

In Figure 10c, an interesting situation arises in PUMA and ERASCA when the number of receivers increases. Due to the increase in the number of receivers, flooding increases, and the overhead increases. This increase in overhead increases the packet drop, link failure, congestion, delay, etc. On the other hand, in EERASCA when the number of nodes increases, the overhead decreases due to multicasting and unicasting. Hence, the richer connectivity among the receivers decreases the overhead, packet drop, delay, congestion and bandwidth, etc., showing performance improvement compared to ERASCA and PUMA by avoiding flooding. In Figure 10, with fewer receivers, multicasting and unicasting are ideal because of less congestion and successful packet transmission to the desired destination with less packet drop. EERASCA shows good improvement related to overhead, throughput, and PDF compared to PUMA and ERASCA where flooding is used. Hence, EERASCA outperform the ERASCA and PUMA in PDF and throughput as the number of receivers are increases. The confidence interval for PDF, overhead and Throughput is shown in Table 4 in the presence of receivers (10–40), which shows the mean value, highest and lowest value.

#### 7.2.3. Scenario 3

**Figure 11:**
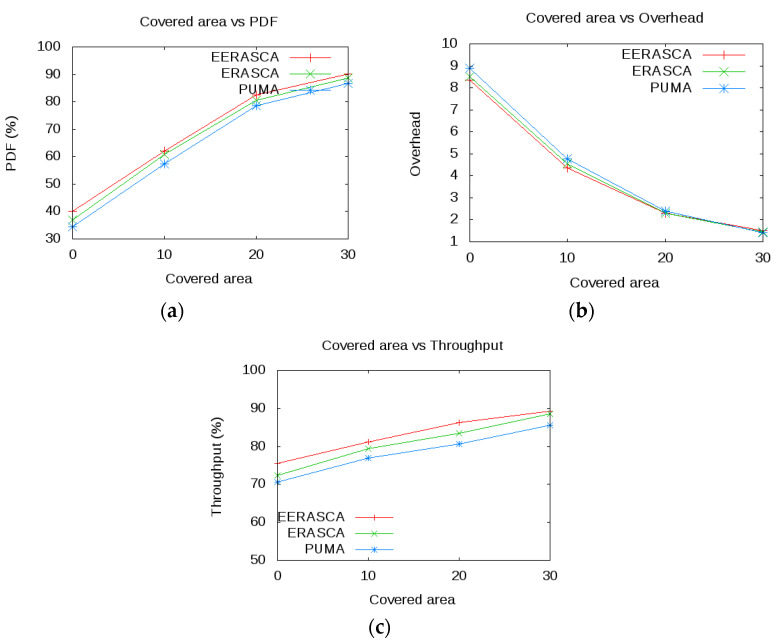
Comparison of Covered Area with PDF, Overhead and Throughput.

Figure 11 shows the PDF, throughput and overhead vs covered area. In 30 × 30 m^2^ terrain dimension with PUMA, ERASCA and EERASCA protocol, EERASCA demonstrates superiority over PUMA, ERASCA within a small, covered area because of the fewer possibilities of packet drop due to link failure. Hence, throughput and packet delivery fraction improve and overhead depreciates. In contrast, with the increase in the covered area, throughput and packet delivery fraction decrease, but its graph is still higher than the PUMA and ERASCA, as shown in Figure 11a,b. The performance further deteriorates when flooding is used. EERASCA shows improved PDF, overhead, and throughput in 11c than PUMA and ERASCA because both PUMA and ERASCA flooding are used. In EERASCA, multicasting is used in a large, covered area, and unicasting is used in a small covered area with a smaller number of nodes. The confidence interval for PDF, overhead and throughput is shown in Table 5 in the presence of covered area (0–30 m^2^), which shows the mean value, highest and lowest value.

## 8. Conclusions

In this paper, the proposed EERASCA protocol is presented based on efficiency and reliability of the sensor network. 

In this paper, unicasting and multicasting are used for data forwarding from small to large network instead of flooding to improve throughput and overhead. The proposed protocol gives promising results in low and medium mobility. However, in high mobility, PUMA and ERASCA performance is better than the proposed protocol. In the proposed protocol, as the nodes increases then throughput, PDF and overhead is improving as compared to PUMA and ERASCA. Similarly, by increasing the coverage area, the number of entry and exit of the node increases. Hence, frequent flooding and reconfiguration occur in PUMA and ERASCA, which further decreases the performance in packet drop, congestion, overhead, PDF and throughput as compared to EERASCA. The protocol is tested in real-time as well as in NS-2 environment, and it outperforms the PUMA and ERASCA in term of throughput, PDF and overhead.

The future work in this paper shows that its performance is not satisfactory, similar to PUMA and ERASCA during high mobility in term of throughput and PDF, which can be improved by improving its clustering mechanism.

## Figures and Tables

**Table 1:** Comparison of ERASCA and Proposed EERASCA Protocol.

Stage No.	Hierarchy of the Paper	Previous/Base Protocol (ERASCA)	Proposed Protocol (EERASCA)
Stage 1	SD Message	Similar	Similar
Stage 2	Connectivity List	Similar	Similar
Stage 3	Core Election Process	Similar	Similar
Stage 4	Mirror Core Election Process	Similar	Similar
Stage 5	Receiver Group Formation	Similar	Similar
Stage 6	Mesh Formation	Similar	Similar
Stage 7	Data Forwarding Process	Similar	Different
Stage 8	Algorithm	Similar	Different
Stage 9	Mode of Communication	Similar	Different

**Table 2:** Parameters of Testbed.

Parameters	
Number of nodes	30
Mobility	4 m/s
Covered area	30 × 30 m^2^
Time of execution	450 s
MAC type	MAC 802.11

**Table 3:** Confidence Interval for PDF, Overhead and Throughput.

Protocol	Mean Interval	Confidence Interval
EERASCA PDF	79.96%	89.10–70.82%
ERASCA PDF	82.02%	85.05–78.99%
PUMA PDF	77.39%	81.33–73.45%
EERASCA Overhead	2.35 byte/s	2.16–2.55 bit/s
ERASCA Overhead	2.47 byte/s	2.34–2.60 bit/s
PUMA Overhead	2.75 byte/s	2.65–2.85 bit/s
EERASCA Throughput	79.26%	89.71–68.82%
ERASCA Throughput	79.20%	86.11–72.30%
PUMA Throughput	77.25	83.10–71.40%

**Table 4:** Confidence Interval for PDF, Overhead and Throughput.

Protocol	Mean Interval	Confidence Interval
EERASCA PDF	90.56%	93.08–88.04%
ERASCA PDF	84.88%	86.08–83.68%
PUMA PDF	79.94%	81.45–78.43%
EERASCA Overhead	2.41 byte/s	2.47–2.36 bit/s
ERASCA Overhead	2.45 byte/s	2.50–2.40 bit/s
PUMA Overhead	2.49 byte/s	2.54–2.44 bit/s
EERASCA Throughput	88.54%	89.70–87.38%
ERASCA Throughput	86.53%	87.83–85.23%
PUMA Throughput	82.55	83.65–81.45%

**Table 5:** Confidence Interval for PDF, Overhead and Throughput.

Protocol	Mean Interval	Confidence Interval
EERASCA PDF	65.18%	93.23–40.12%
ERASCA PDF	62.70%	88.54–36.86%
PUMA PDF	60.62%	86.78–34.45%
EERASCA Overhead	4.93 byte/s	8.36–1.50 bit/s
ERASCA Overhead	4.98 byte/s	8.51–1.45 bit/s
PUMA Overhead	5.175 byte/s	8.90–1.45 bit/s
EERASCA Throughput	82.39%	89.23–75.55%
ERASCA Throughput	80.44%	88.54–72.34%
PUMA Throughput	78.10	85.55–70.65%

## Data Availability

The simulation files/data used to support the findings of this study are available from the corresponding author upon request.
